# Study on Different Water-Based Binders for Li_4_Ti_5_O_12_ Electrodes

**DOI:** 10.3390/molecules25102443

**Published:** 2020-05-24

**Authors:** Christina Toigo, Catia Arbizzani, Karl-Heinz Pettinger, Maurizio Biso

**Affiliations:** 1 Department of Chemistry “Giacomo Ciamician”, Alma Mater Studiorum Universitá di Bologna, Via F. Selmi 2, 40126 Bologna, Italy; catia.arbizzani@unibo.it; 2 Technology Center for Energy, University of Applied Sciences Landshut, Am Lurzenhof 1, 84036 Landshut, Germany; karl-heinz.pettinger@haw-landshut.de; 3 Solvay Specialty Polymers, 20021 Bollate, Italy; Maurizio.biso@solvay.com

**Keywords:** lithium ion battery, anode, water-based PVDF binder, lithium titanium oxide, sodium alginate, C-rate capability, stability test, LTO, environmentally friendly coating

## Abstract

In this study, Li_4_Ti_5_O_12_ (LTO) electrodes with different types of water-soluble binders are successfully coated upon aluminum foil. Electrodes containing solely sodium alginate (SA) as a binder or a mixed PVDF/carboxymethyl cellulose (CMC) binder show the most stable performance in 1 M LiPF_6_ in EC/DMC 1:1 in half cell vs. Li, with respect to cycle stability over 100 cycles at 1 C. The electrodes processed with a mixture of PVDF/SA show considerable fading and slightly worse values for rate capability. Each one of the different binders used is eco-friendly, and the whole processing can be performed without the use of organic solvents. Further advantages covering the whole production and recycling process, as well as safety issues during operation, encourage deeper research in this area.

## 1. Introduction

In recent times, there have been discussions about the safety and (environmental) sustainability of lithium ion batteries (LIBs). It is obvious that battery performance is no longer the only critical feature, and a change in focus towards a broader definition of a “good battery” can be observed. Intensive research is also being carried out on so-called inactive materials such as separators, conductive agents and binders, and they are being optimized for different applications. One possible way to manufacture battery cells is by a completely water-based route without the use of organic solvents. Graphite is likely the most widely used anode material for LIBs due to its high theoretical capacity and long cycle life. Graphite struggles with the formation of a solid electrolyte interphase (SEI) caused by the electrolytes’ instability in the presence of lithiated graphite. In the course of several cycles of charging and discharging, the fragile and non-uniform SEI film will crack because of surface defects and re-form [1]. Silicon has also been under investigation as another possible anode material, but its massive volume expansion during cycling hinders its commercialization in the near future [2] and leads to a bad cycling stability [3]. In this study, we focus on spinel Li_4_Ti_5_O_12_ as an attractive alternative anode material. It has an Li insertion voltage of 1.55 V vs. Li^+^/Li, where the commonly used electrolytes are thermodynamically stable. Combined with the fact that it exhibits a good C-rate capability, the fast lithium intercalation and high cycling stability are the most important advantages of Li_4_Ti_5_O_12_ (LTO) [4]. In contrast to silicon, LTO is considered as a zero-strain material, with a volume increase of 0.2% for lithiation [5]. 

Several studies have examined the performance of Li_4_Ti_5_O_12_ as an anode material for lithium ion batteries [4,6,7,8,9,10,11,12]. Only some of them concentrate on new water-soluble and environmentally friendly binders, despite the well-known and well-established carboxymethyl cellulose (CMC). A PEG (polyethylene glycol)-based binder was investigated by Tran et al., with the resulting LTO delivering 4.2 mAh/cm² at C/2 rate [12]. With the acrylic aqueous binder LA132 studied by Karuppiah et al. [6], LTO delivered a total theoretical capacity of 175 mAh/g at C/2 rate after cycling up to 20 C. Carvalho et al. investigated guar gum and pectin, which showed higher discharge capacities up to 5 C compared to a standard formulation using CMC [9]. De Giorgio et al. studied sodium alginate (SA) as a possible binder for LTO anodes displaying high specific capacities in the range of 145 mAh/g at 10 C [4].

No reports on the use of water-based PVDF binder for LTO electrodes have been found whereas many studies on cellulose-like binders, such as CMC and PVDF binder in organic solvent, have been performed. The use of organic solvents such as NMP is a topic worth discussing, as many drawbacks concerning safety and ecological reasons have yet to be resolved. 

**Figure 1:**
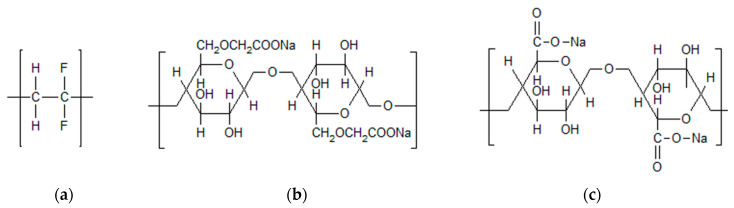
Chemical structures of (**a**) PVDF, (**b**) sodium carboxymethyl cellulose and (**c**) sodium alginate.

**Figure 2:**
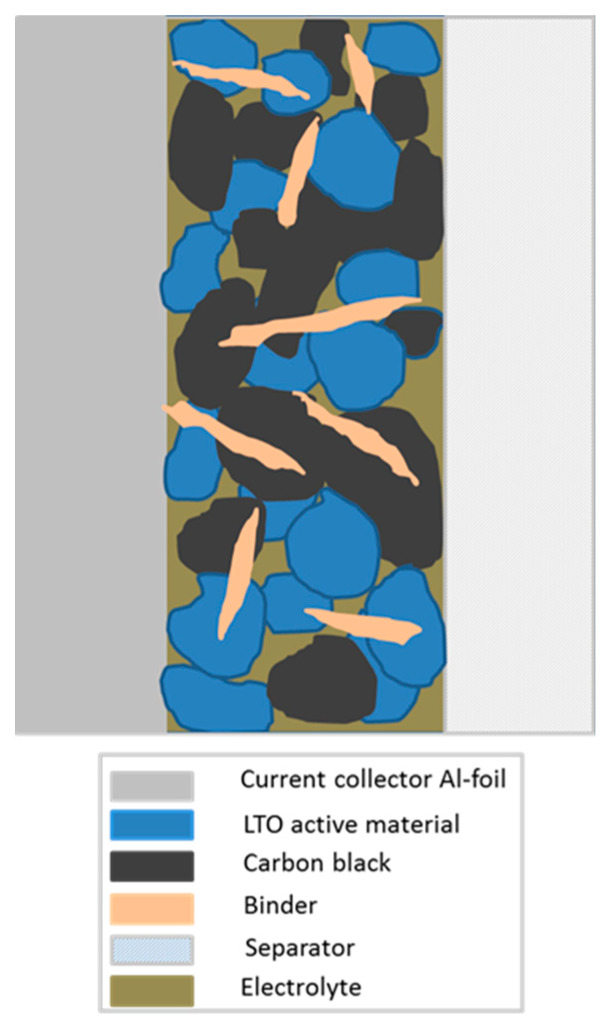
Cross-section scheme of electrode setup.

Each one of the binders used in the present study (as shown in Figure 1) is eco-friendly, and the whole processing can be performed without the use of organic solvents. Though binders themselves are electrochemically inactive components, their chemical and physical nature definitely affects battery performance, especially capacity retention and cycle life [13]. Key objectives of binders in battery electrodes are of course enabling a connection within the active material as well as a connection to the current collector (see Figure 2), the maintenance of the electrodes’ physical structure and the formation of an electric network between active material and carbon [14]. In order to be able to perform all of these tasks, binders must fulfill several requirements. The most important are a good electrochemical stability, good adhesion properties and a high strength. The investigated binders are sodium carboxymethyl cellulose (CMC), sodium alginate (SA) as well as a novel PVDF binder in aqueous dispersion (PVDF), which has been introduced by Solvay Specialty Polymers, Italy, and has recently been investigated as a binder for graphite anodes by our group [15]. PVDF binders that are processed in organic solvents show good reversible capacity and provide electrochemical stability, reversibility, good wettability and resistance to oxidation and chemicals [16]. Therefore, they are widely used for the preparation of anodes in lithium ion batteries. The main drawback that is listed in nearly every publication [5,6,9,12,13,17,18,19,20,21,22] is the use of a flammable and toxic solvent such as NMP. This can be overcome by the use of PVDF in an aqueous dispersion, thus preserving the advantageous properties mentioned above.

What is apparent at first glance is the great difference between the PVDF structure and the structures of CMC and SA. SA, as a natural polysaccharide extracted from brown algae, contains carboxylic groups in each of the polymeric units [23]. Similarly, CMC is a cellulose derivate with carboxymethyl groups substituting some of the hydroxyl groups of cellulose [11]. These groups enable a great number of hydrogen bonds between the binder and the active electrode material, therefore favoring particle cohesion [13,21]. Its strong hydrophilicity and stable internal network structure are some more advantages of CMC [24]. Different studies have been performed on the swelling ability of SA. Kovalenko et al. found no detectable swelling of SA films, with its behavior similar to that of CMC binder occurring (in contrast to PVDF, which has a change in thickness of 17% [15] up to 20% [23]), whereas Samanta et al. claim a high swelling ability of SA hydrogels [25]. In contrast to the binders enabling hydrogen bonds, PVDF interacts with the active material via weak van der Waals forces, only on account of the –C-F functional group [22]. It fails to accommodate large changes in spacing between the particles, which was discovered for both lithium ion batteries and silicon anodes [26].

Another difference between the bio-based binders and PVDF is their thermal stability. Cuesta et al. measured the thermal stability of binders via TGA and found that all tested binders proved to be thermally stable at least up to 200 °C, which is roughly the onset temperature of the first decomposition stage for SA. CMC turned out to be slightly more stable with a decomposition onset temperature of 240 °C, whereas PVDF showed a much higher thermal stability up to 400 °C [21].

Thus, we suggest that a combination of water-based PVDF binder and either CMC or SA should lead to the best results concerning cyclability and stability of the electrode, by taking advantage of both types of binders.

Here, we report the use and combination of the different water-based binders mentioned above to prepare LTO electrodes, which were tested in half-cell configuration. The electrodes were successfully coated on aluminum. The prepared electrodes were tested mechanically and electrochemically in 1 M LiPF_6_ in EC/DMC 1:1 in half cell vs. Li, and showed an overall stable coating combined with a good cycling stability and power capability up to 5 C.

## 2. Results and Discussion

Electrodes with LTO as the active material were prepared and tested using different kinds of binders with the same percentage of active material (90%), conductive carbon (6%) and an overall percentage of 4% for the binder(s), as shown in Table 1. The homogeneous electrode film was coated upon an Al/C current collector. 

**Figure 3:**
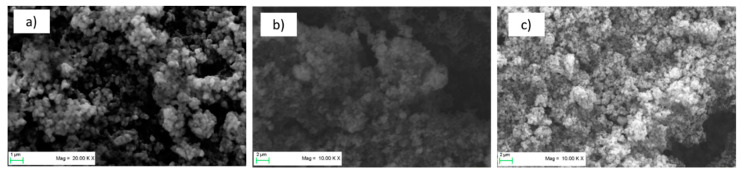
SEM images of the three types of pristine electrodes: (**a**) Li_4_Ti_5_O_12_ (LTO)-sodium alginate (SA), (**b**) LTO-PVDF-carboxymethyl cellulose (CMC) and (**c**) LTO-PVDF-SA.

Figure 3 shows SEM images of the three types of pristine LTO electrodes. The electrodes appear well-mixed and show a homogeneous distribution of the materials. No agglomerates of materials are visible. Although the electrodes showed very good mechanical stability during processing (punching and placing in half-cell apparatus), this was tested further with a peeling test. 

**Figure 4:**
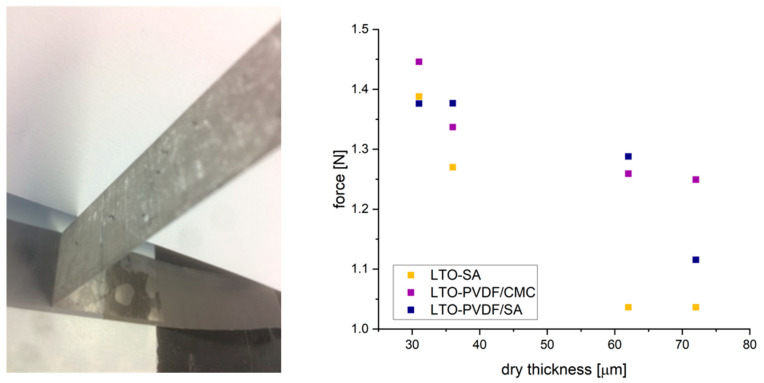
Setup and results of peeling test.

Peeling tests show the weaknesses of pure SA binder in comparison to PVDF/CMC and PVDF/SA, as can be seen in Figure 4. Both binder systems perform remarkably better at dry thickness of more than 35 microns. In general, a reduction of the required peeling force is observed with an increase in the thickness of the electrode, which is a well-known phenomenon based on slower drying since the slurry contains a higher fluid fraction [27]. The addition of PVDF binder improves the adhesive and cohesive binding properties, leading to more mechanically stable electrodes.

**Figure 5:**
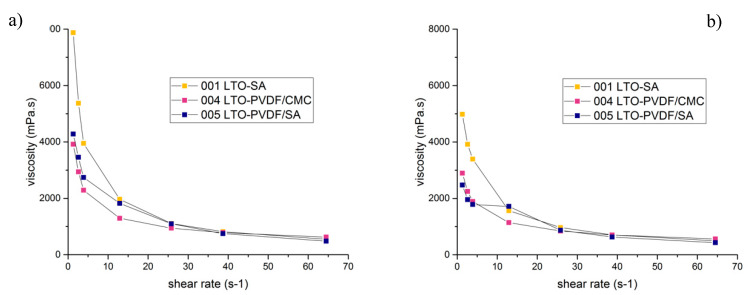
Viscosity as a function of the shear rate for different slurries: (**a**) directly after mixing and (**b**) after 24 h.

**Figure 6:**
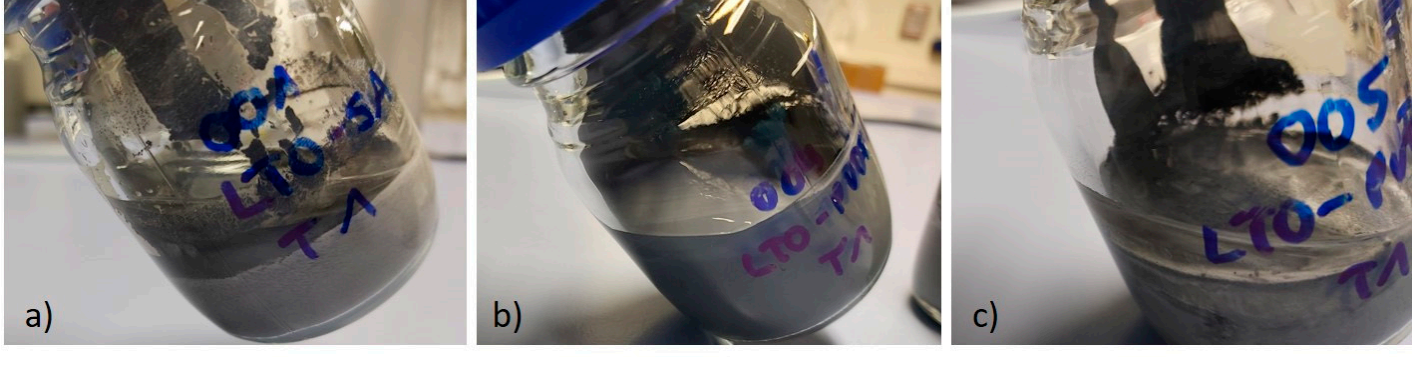
Difference between the slurries 7 days after mixing with: (**a**) LTO-SA, (**b**) LTO-PVDF/CMC and (**c**) LTO-PVDF/SA binder systems.

To evaluate the slurry stability, a viscosity test was performed for all slurries in different time ranges: after mixing, 24 h after mixing and seven days after mixing. The results are displayed in Figure 5 and show some very interesting properties. The viscosity of SA-slurries was higher than any of the PVDF-mixtures for shear rates between 1 and 10 s^−1^. Quite similar viscosity values for all slurries are reached for shear rates above 10 s^−1^ up to 65 s^−1^. One interesting aspect is the difference in viscosity after 24 h: it decreased for all different types of slurry and it should be noted that this effect is mostly visible at low shear rates. The SA-containing slurries displayed a much greater change in viscosity than those without SA, probably caused by a slight de-mixing, as can be seen in Figure 6. In general, a higher viscosity is known to prevent LTO particles from sedimentation and aggregation during electrode fabrication when water is evaporating. This leads to a high uniformity of the slurry [23]. The higher viscosity of the SA-slurries at low shear rates appears as an indicator for a good network structure, but the effect is relativized at higher shear rates, which are necessary for coating. Concerning the coating procedure, a higher slurry viscosity requires lower coating speeds in order to establish a better contact between the slurry and the current collector foil. Furthermore, alginate macromolecules are much more polar than the CMC polymer chains, which can ensure better interfacial interaction between the polymer binder and the particles [23]. On the contrary, the results of the peeling tests show a very different picture, most likely caused by high shear rates during coating.

**Figure 7:**
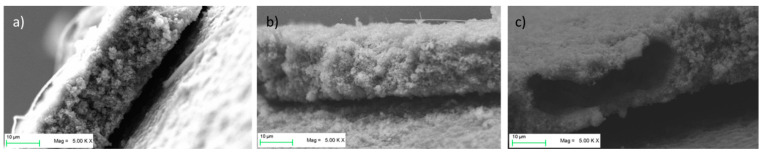
SEM cross-section images of electrodes after 80 cycles: (**a**) LTO-SA, (**b**) LTO-PVDF/CMC and (**c**) LTO-PVDF/SA.

**Figure 8:**
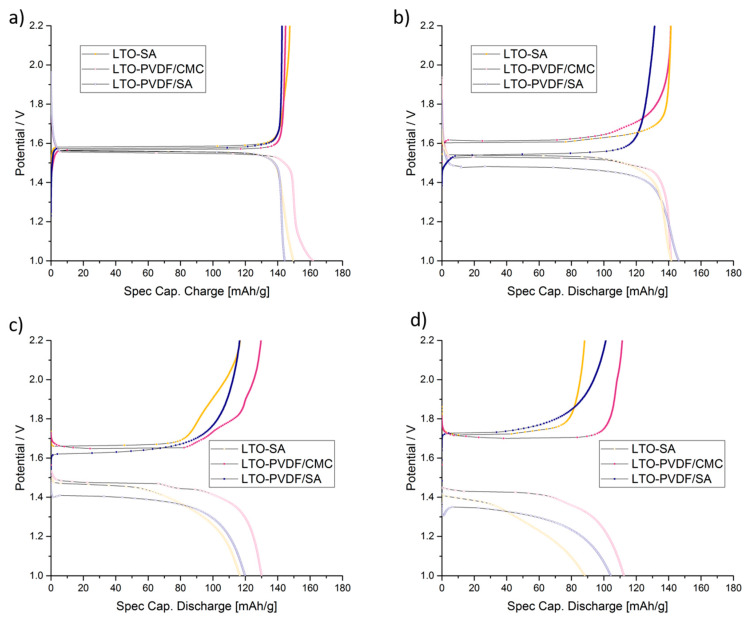
Charge–discharge profiles of LTO anodes at: (**a**) 0.33 C, (**b**) 1 C, (**c**) 5 C and (**d**) 10 C.

**Figure 9:**
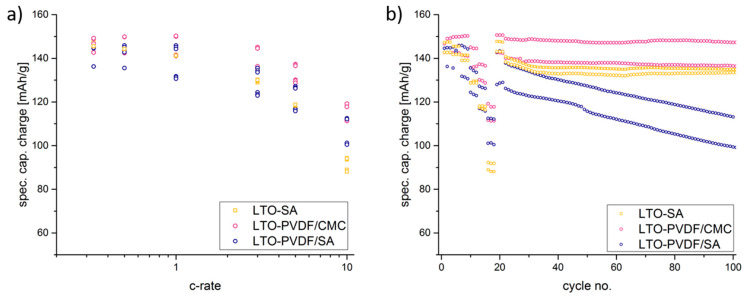
Electrochemical performance during C-rate test (**a**) and charge capacities for each of 2 exemplary electrodes (**b**).

Figure 7 shows the SEM images of the electrodes that sustained electrochemical tests. While LTO-SA and LTO-PVDF/CMC electrodes show a homogeneous distribution of the particles, LTO-PVDF/SA displays a huge hole in the dimensions of about 8 × 20 μm. Holes of this dimension were only found for this recipe and are suspected to be caused by insufficient degassing before the coating step, combined with a very active swelling behavior of both PVDF and SA binders, leading to a worse interconnection between active material and binder up to a complete contact loss within the electrode. Charge-discharge profiles of the different electrodes show a weak performance of the LTO-SA binder system at high C-rates (see Figure 8). Furthermore, we assume local pH incompatibilities between PVDF and SA also led to the strong fading behavior in electrochemical tests (see Figure 9b). The SEM images also display residues of the glass fiber separator on the electrode surface due to the tight packing within the half-cell arrangement. 

**Figure 10:**
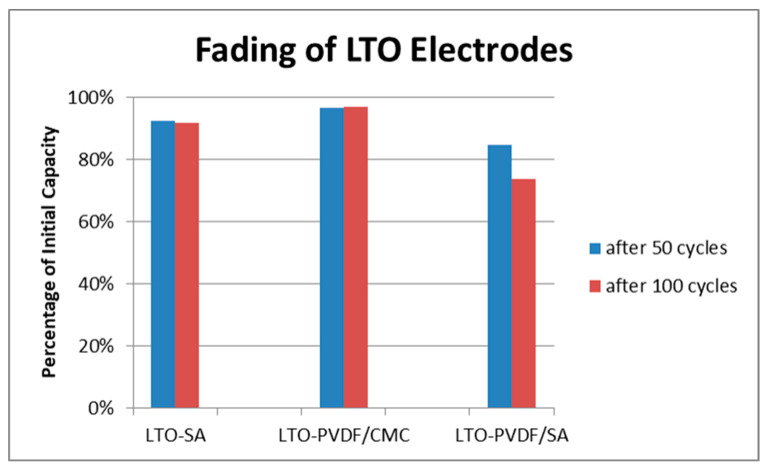
Fading of LTO electrodes with different binders.

Figure 8 shows the charge–discharge curves of LTO anodes at different C-rates in the voltage range of 1.0–2.2 V, where 1C equals the theoretical capacity of 175 mAh g^−1^. Furthermore, the delivered discharge capacities of LTO anode half cells are clearly visible and show up with 162 (92.6% of the theoretical capacity), 149 (85.1%) and 145 (82.9%) mAh g^−1^ at 0.333 C-rate with PVDF-CMC, SA and PVDF-SA binders, respectively. When charged and discharged repeatedly, the different water-based electrodes show stable capacities up to 5 C (Figure 9a), but a quite considerable fading occurs when the PVDF/SA binder (Figure 9b and Figure 10) is observed. Figure 9b shows a combined C-rate and cycling test starting with each three galvanostatic cycles at 0.33/0.5/1/3/5 and 10 C, followed by 80 cycles at 1 C. Indeed, the binder containing PVDF/SA retrieves significant fading at 84.8% of the initial capacity for 50 cycles and 73.9% for 100 cycles, taking the very first one as a reference cycle. Having a look at Figure 7, one can assume that the bad electrochemical behavior is caused by the poor electrode structure and the associated bad electrical conductivity, probably resulting in a contact loss between electrode and current collector. 

From the electrochemical point of view, the combination of PVDF/CMC produces electrodes that deliver the best results both for cycling stability and C-rate capability. This result means that some reasons for the different behavior of PVDF/CMC and PVDF/SA have to be considered. On the one hand, one might expect a similar behavior due to the fact that CMC and SA have quite a similar structure and are combined with the same fluoropolymer. On the other hand, one has to consider that for CMC, the presence of carboxymethyl groups is responsible for aqueous solubility. It is a weak polyacid exhibiting pH-dependent dissociation forming anionic carboxylate functional groups [28]. With an increasing percentage of SA within a mixture, the swelling ratio increases, probably attributed to an increase in electrostatic repulsive force in the network because of negatively charged carboxylate functional groups [25], which can lead to a less sufficient binder–particle connection. The stiffness and swelling ability of SA also depends on the sequence and composition of the alginate chain due to the differences in the stereochemistry of mannuronic and guluronic acid monomers, as well as on the electrostatic interactions with cations in solution [29]. Due to a higher polarity of SA compared to CMC polymer chains, a hindering of both binders in the combination PVDF/SA is another possible mechanism leading to the bad electrochemical results of this recipe [23].

Therefore, we suggest a negative interaction of PVDF with SA regarding their swelling behavior. The medium crystallinity of the used PVDF binder (30% of polymer chains) means that 70% of the binder’s polymer chains are in amorphous state and are therefore able to perform swelling. In these swollen regions, the electrolyte can easily penetrate into the composite electrode and ensure a good ion transport. This leads to the suggestion that a crystallinity of 30% results in smaller proportions of the binder having the ability to share electrical conductivity with the active material, since swelling only occurs in amorphous regions [14,15]. 

The high electrolyte uptake of PVDF due to its flexible linear chains [30] and long-term soaking of the electrolyte during battery storage and cycling [22], combined with the good swelling ability of SA in water, leads to worse electrochemical results. Firstly, it can hinder the interaction of the binder with the active material and secondly, cause the particles to lose contact within operation of the battery, leading to a lower conductivity and a degradation of battery performance. 

In contrast, a combination of PVDF and CMC shows very promising results. PVDF with its good wettability toward polar electrolyte solutions easily performs the Li ion transport [31]. Furthermore, the strong hydrogen bonding of the carboxyl and hydroxyl groups in CMC with the active material and the current collector seems to be a perfect complement for the PVDF binder, which only forms weak hydrogen bonds [11]. 

The high and nearly linear fading of LTO-PVDF/SA (as can be seen in Figure 9b) can also be a hint for a bad adhesion of the electrode upon the collector foil, thus resulting in worse capacity cycle by cycle. Moreover, a kinetic difference is indicated between the PVDF and CMC binders; electrodes containing solely CMC binder have a much lower charge transfer resistance, a lower apparent activation energy and a lower apparent diffusion activation energy than electrodes containing PVDF binder [3].

## 3. Materials and Methods 

### 3.1. Electrode Preparation

Electrodes were prepared with commercial lithium titanium oxide (90 wt. %, LTO Toda Kogyo, Hiroshima, Japan) and conductive carbon (6 wt. %, Super C65, Timcal, Bironico, Switzerland). Sodium alginate (SA, Sigma-Aldrich, Munich, DE, USA), CMC (MAC 200 HC, Nippon Paper Industries, Chiyoda, Japan) and a water-based PVDF binder (Solvay Specialty Polymers, Italy) were used in different weight ratios to obtain a total binder amount of 4%. The used emulsion of water-based PVDF binder was stored at room temperature and homogenized before usage. Binder crystallinity was determined by DSC and found to be 30%. Deionized water was used as a solvent for anode preparation. A quantity of 1 M LiPF_6_ in ethylene carbonate and vinylene carbonate (Selectilyte RD1001, BASF) was used as the electrolyte, and a glass-fiber separator (Sartorius) was used for the half-cell setup. The electrode formulations are reported in Table 1. 

The processing was carried out by mixing LTO active material (90%), conductive carbon (6%) and the different amounts of binder with deionized water to produce a solids content of 40% in a high-speed dissolver (Dispermat CV3-plus, VMA-Getzmann GmbH, Reichshof, Germany). The mixing was performed at 2000 rpm for 120 min. Viscosity of anode slurries was measured with a Brookfield DV3T viscometer (Middleboro, MA, USA) and resulted in values between 3917 and 7873 mPa·s. More viscosity tests were performed at 1 and 7 days after mixing. The slurries were manually casted on a carbon-coated Al-foil current collector and dried in a two-step drying tunnel at a temperature range of 90–100 °C. The average areal capacity of the electrodes was 0.35–0.50 mAh/cm². The water-processed electrodes were cut into round pieces with a diameter of 12 mm and dried at 110 °C for 24 h under vacuum. The Swagelok setup in the form of a three-electrode arrangement was assembled in an argon-filled glove box (MB20, H_2_O and O_2_ <1 ppm). For both reference and counter electrodes, lithium metal was used. A Zeiss Merlin Compact Field Emission Scanning Electron Microscope was used to take micrographs of both the fresh and the cycled electrodes. In order to remove electrolyte residues, the cycled electrodes were rinsed before analysis.

### 3.2. Mechanical Characterization

Peeling tests were performed by cutting specimens from coating thicknesses of 31, 36, 61 and 72 microns with a size of 50 × 80 mm, and stuck with a double-sided adhesive tape upon the supporting surface. A 19-mm adhesive tape was pressed upon the coating, and we then attempted to peel it off with a Peel-off Force Special Test Stand (TPE 50, Sauter GmbH, Balingen-Frommern, Germany) orthogonal to the supporting surface. The maximum resulting force is recorded as peel-off force.

Bending tests were performed by bending the coated foil around a roll with a diameter of 0.8 cm. Adhesive or cohesive breaking was evaluator assessed. 

### 3.3. Electrochemical Characterization

The electrochemical characterization was performed with a battery tester (CTS-lab, BaSyTec, Asselfingen, Germany), using galvanostatic (CC) and potentiostatic (CV) modes for charging and CC mode for the discharging step. For half-cell measurements, the voltage range was adjusted to 1.0–2.2 V. Formation was done by three cycles of 0.33 C, which was calculated by the active material weight and using a theoretical capacity of LTO of 175 mAh/g. C-rate testing was performed for each of the three galvanostatic cycles at 0.33/0.5/1/3/5/10 C, followed by a cycling test at 1C.

## 4. Conclusions

The tested electrodes were prepared by a completely water-based, environmentally friendly method. LTO anodes with SA, PVDF/CMC and PVDF/SA binder systems were successfully coated upon aluminum foil using conventional slurry and electrode coating techniques. The prepared electrodes were tested mechanically (peeling test and bending test) and electrochemically in half-cell configuration. LTO anode with PVDF/CMC binder system showed an overall highly stable coating. Neither cycling stability nor rate capability resulted in significant differences for C-rates up to 1C, whereas for high C-rates the advantages of PVDF/CMC binder systems showed up clearly. This promising processing of completely water-based binder systems shows a possible way for the production of ecologically safer batteries.

## Figures and Tables

**Table 1:** Formulation of LTO electrodes.

Composition	LTO-SA	LTO-PVDF/CMC	LTO-PVDF/SA
LTO active material	90	90	90
Super C	6	6	6
Sodium alginate	4	-	2.67
CMC	-	2.67	-
PVDF	-	1.33	1.33
